# The Burden of Pediatric Atopic Dermatitis: Quality of Life of Patients and Their Families

**DOI:** 10.3390/jcm13061700

**Published:** 2024-03-15

**Authors:** Inga Kisieliene, Antanas Mainelis, Odilija Rudzeviciene, Matilda Bylaite-Bucinskiene, Andreas Wollenberg

**Affiliations:** 1 Clinic of Infectious Diseases and Dermatovenereology, Vilnius University, 08411 Vilnius, Lithuania; 2 Faculty of Mathematics and Informatics, Vilnius University, 03225 Vilnius, Lithuania; 3 Clinic of Children’s Diseases, Faculty of Medicine, Vilnius University, 03101 Vilnius, Lithuania; 4 Department of Dermatology and Allergy, Augsburg University Hospital, 86156 Augsburg, Germany; 5 Department of Dermatology and Allergy, Ludwig-Maximilian University of Munich, 80539 Munich, Germany; 6 Comprehensive Center for Inflammation Medicine, University of Luebeck, 23562 Luebeck, Germany

**Keywords:** atopic dermatitis, children, quality of life, the Family Dermatology Life Quality Index, the Patient-Oriented Eczema Measure

## Abstract

**(1) Background:** Atopic dermatitis (AD) is one of the most common inflammatory skin conditions, thus having a significant impact on the quality of life (QoL) of patients and their families. We performed a survey to gather more data to help describe the burden of AD in Lithuania and to help expand the treatment plan to this important aspect of the disease. **(2) Methods:** A cross-sectional study was conducted involving healthy and AD pediatric patients. The assessment instruments used were the Patient-Oriented Eczema Measure (POEM), QoL, and original questionnaires (the original questionnaire was designed by the authors to determine the demographics, medical history, and treatment methods of the respondents). **(3) Results:** This study included 302 participants in total: 247 AD patients (51% boys) and 55 non-AD patients (51% boys). The mean age for AD patients was 6.8 ± 4.4. years, and this was 10.5 ± 3.1 years for the control patients. A significant difference was found between the QoL questionnaire scores and the Family Dermatology Life Quality Index (FDLQI) score for the atopic dermatitis group (QoL: 6.3 ± 5.6; FDLQI: 7.1 ± 6.9) and controls (QoL 0.5 ± 1.1; FDLQI 2.1 ± 5.9) (*p* = 0.000). The mean QoL questionnaire score for severe AD was 14.3 ± 6.2 (very large effect), that for moderate AD was 6.9 ± 4.4 (moderate effect), and that for mild AD was 4.4 ± 4.2 (small effect) (*p* = 0.000). **(4) Conclusions:** Our study revealed a moderate effect of AD on dermatology-related QoL in patients and their families. It has been shown that increased disease severity was associated with a greater impairment of QoL in both patients and patient’s parents. The burden of AD in children and their parents is considerable and should be taken into account in the management of atopic dermatitis.

## 1. Introduction

Pediatric atopic dermatitis (AD) is a common skin condition affecting 12–15% of children in early childhood [1], and there is considerable evidence that its prevalence is increasing. In a study using International Study of Asthma and Allergies in Childhood (ISAAC) standardized questionnaires carried out in Kaunas, Lithuania, in 1994–1995 and 2001–2002 among schoolchildren aged 6–7 years, the prevalence of atopic dermatitis was found to have increased from 1.4% to 3.5%. The reason for this increase remains unclear; genetic, immunological and environmental factors may be important [2]. Symptoms vary from individual to individual, and there is a tendency for it to remit and exacerbate unexpectedly. The skin lesions, which are usually accompanied by severe pruritus, include infiltrated erythema, erythema with erosions caused by scratching, lichenified areas, and pruriginous papules and nodules. The symptoms of AD affect the psychological and mental health of patients, mainly due to the chronic relapsing disease course, intense pruritus, the unaesthetic appearance of lesions, and sleep disturbance [3,4].

The symptoms of atopic dermatitis as well as the demands of treatment often contribute to a significant impact on patient quality of life (QoL). This quality of life impairment may also extend to caregivers, partners, and close family members of atopic dermatitis sufferers [5]. Adequate treatment of AD is essential for the successful management of the disease and for improving patients’ quality of life. Mild-to-moderate AD is treated with emollients, topical anti-inflammatory and antimicrobial agents, and phototherapy. Meanwhile, severe AD requires systemic treatment. Three substance classes are now available for systemic anti-inflammatory treatment: conventional immunosuppressants, biologic agents, and Janus kinase (JAK) inhibitors. Systemic glucocorticosteroids should only be used in exceptional cases and for short periods (from a few days to three weeks) to treat an acute flare [3,6]. Cyclosporine is approved for the short- and medium-term treatment of severe atopic dermatitis in patients aged 16 and older, and should not be used for more than two years, preferably as an interval therapy every few months. Methotrexate or azathioprine can be used off-label in individual cases for longer-term immunosuppression. Dupilumab and tralokinumab are monoclonal antibodies for subcutaneous injection. Dupilumab binds the alpha subunit of the IL-4 receptor, blocks IL-4 and IL-13 signaling pathways, and is approved for use from age 6 onward. Tralokinumab binds IL-13 and is currently approved for the treatment of moderate-to-severe atopic dermatitis from age 12 onward. Three JAK inhibitors, baricitinib, upadacitinib, and abrocitinib, have been approved to date for the treatment of moderate-to-severe atopic dermatitis in adults [3,6]; baricitinib is approved for pediatric AD patients2 years old and above [7], and upadacitinib is also approved for children aged 12 and above. The new generation of systemic drugs have high efficacy rates and have been shown to improve quality of life. Quality of life is a very important part of mental health, and its assessment is already included as a factor in the choice of treatments in treatment guidelines of skin diseases [3,6].

QoL measurement has become an integral aspect of monitoring disease and intervention efficacy across the dermatology field. Various recent studies have found a direct relationship between the dermatology life quality index (DLQI) and disease severity [8,9,10]. QoL measures can provide valuable insights into the impact of disease, which can be used for comparison between individuals with any disease. Measuring QoL can be a powerful political tool providing information from a patient perspective on the health impact of different diseases. This is particularly important when arguing for vital resources, especially in skin disease, which is not generally considered to have much of a life impact, as judged by non-dermatologists and the general public [11]. There are few data on how atopic dermatitis affects the DLQI of children and their family members in Lithuania. We performed a survey to gather more data from patients and caregivers to help describe the disease burden. Our study aims to evaluate the impact of atopic dermatitis on the QoL of patients and family members, and to identify its relationships with disease severity. The results of this study will equip clinicians with the knowledge to actively address the burden of AD on patients and their caregivers, with the hope of improving their QoL through an integrated treatment plan.

## 2. Materials and Methods

A cross-sectional sample of children with atopic dermatitis who came to a dermatovenereologist consultation at Vilnius University Hospital Santaros klinikos Clinic of Children’s Diseases were invited to participate in the survey (between 1 January 2020 and 31 December 2022). Patients were recruited via consecutive sampling based on their clinic appointment. Eligibility criteria for the atopic dermatitis group were defined as individuals living in Lithuania, younger than 18 years, who were clinically diagnosed with AD according to Hanifin and Rajka’s diagnostic criteria for AD. The control group was composed of patients (<18 years), with no history of eczema or psoriasis, who presented for a general skin examination. The exclusion criteria included any severe exacerbated chronic, congenital, or oncological disorders that could affect the quality of life of patients. Those who were not able to understand the questionnaire, those who were illiterate, and those who declined participation were excluded. 

Background and quality of life characteristics were obtained through patient and parental questionnaires. Participants had to fill in an original questionnaire, which included 45 questions, created by the authors, for demographic and epidemiological data. The format of the questionnaire included multiple-choice questions, yes/no questions, Likert scales, and open-ended questions. In addition, to evaluate the dermatology life quality index (DLQI) of children with AD, the following questionnaires were used: for children up to 5 years, the Infant’s Dermatitis Quality Life Index (IDQOL) was used; for children between 5 and 15 years old, the Children’s Dermatology Life Quality Index was used (CDLQI); for children 16 years or older, the Dermatology Life Quality Index (DLQI) was used; and to evaluate QoL for parents, the Lithuanian language version of the Family Dermatology Life Quality Index (FDLQI) was used. Questionnaires were chosen for several reasons: they are validated, are available in Lithuania, have numerous language versions that enable a comparison of the results with those of other authors, can be applied for different skin conditions, and enable comparisons to be made between AD and other dermatoses. 

DLQI questionnaires are self-administered, easy, and user-friendly questionnaires with an average completion time of 126 s. They consist of 10 questions concerning patients’ perception of the impact of skin diseases on different aspects of their QoL over the past week. The questions in the DLQI are classified into 6 heading items: symptoms and feelings (questions 1 and 2), daily activities (questions 3 and 4), leisure (questions 5 and 6), and personal relationships (questions 8 and 9), with a maximum score of 6 for each item, work and school (question 7), and treatment (question 10), with a a maximum score of 3 for each item [12]. In the analysis of the QoLI questionnaires, 5 questionnaires in the control group were excluded due to improper completion. 

The severity of AD was measured using the Patient-Oriented Eczema Measure (POEM) questionnaire. This is a simple, valid, understandable tool for monitoring disease severity in children and adults with atopic eczema that was originally developed to help readdress the imbalance between physician and patient-based outcome measures in eczema research. The POEM assesses the frequency of 7 atopic dermatitis signs and symptoms in the past week, including skin manifestations, itch, and sleep disturbance. Scores range between 0 and 28, and higher scores indicate higher levels of disease activity; 0 to 2 represent clear or almost clear, 3 to 7 represent mild eczema, 8 to 16 represent moderate eczema, 17 to 24 represent severe eczema, and 25 to 28 represent very severe eczema [13].

All statistical analyses were performed using the R (v. 4.0.4) program package. The mean, standard deviation (SD), quartiles (Q1 and Q3), median, and available number of observations of the quantitative variables are presented. Categorical variables are presented as absolute amounts and the percentages. To test hypotheses between the two groups in a comparison of the quantitative variables, Student’s *t*-test or a nonparametric Mann–Whitney U test was used as appropriate. To test hypotheses between more than two groups in a comparison of the quantitative variables, a one-way analysis of variance (ANOVA) or nonparametric Kruskal–Wallis test was used as appropriate. Normality was tested using the Shapiro–Wilks test. To test hypotheses for a between-group comparison of the categorical variables, Pearson’s Chi-Square test or Fisher’s exact test was used as appropriate. A *p*-value less than 0.05 was considered significant.

## 3. Results

This study included 302 children in total, and their parents/caregivers: 247 children who were AD patients (125 boys and 121 girls) and 55 non-AD patients (28 boys and 27 girls). Table 1 describes the personal characteristics of the patients and their parents/caregivers.

The mean age for AD patients was 6.8 ± 4.4 years, and this was 10.5 ± 3.1 years for the control patients. In total, 189 (77%) of AD patients were diagnosed with a disease before the age of 1 year and 218 (89%) were diagnosed before the age of 5 years. Furthermore, 169 (69%) in AD group and 24 (47%) in the control group had a positive family history of atopy (first-degree relatives had/have at least one of the following conditions: atopic dermatitis, rhinoconjunctivitis, bronchial asthma, or food allergy) (*p* = 0.048). In total, 61% of the AD group had at least one atopy disease: 67 (29%) had allergic rhinoconjunctivitis (*p* = 0.022), 99 (43%) had a food allergy (*p* < 0.001), 28 (12%) had allergic asthma (*p* = 0.188), and 13 (5%) were diagnosed with all three atopic diseases. 

A significant difference was found between the QoLI score and FDLQI score for the atopic dermatitis group (QoLI: 6.3 ± 5.6 points; FDLQI: 7.1 ± 6.9 points) and controls (QoL index 0.5 ± 1.1 points; FDLQI 2.1 ± 5.9 points) (*p* = 0.000). Detailed results of the impact of atopic dermatitis on children and family members’ QoL are shown in Table 2 and Table 3. 

Analyzing details of the QoLI questionnaires, we found that children with atopic dermatitis have a great impact on all aspects of life, especially on symptoms and feelings (Q1 and Q2), and treatment (Q10). Healthy patients have no impact on daily activities (Q3 and Q4), leisure (Q5 and Q6), work and school (Q7), or personal relationships (Q8) (Table 2). AD significantly affects children’s friendships (Q3) and activities (Q4, Q5, Q6, and Q7) compared with those of the control subjects.

The mean FDLQI score for AD patients was 7.1 ± 6.9, indicating a moderate effect on the QoL of the patient’s family, and the mean FDLQI score for control patients was 2.1 ± 5.9, representing a low impact on the family’s QoL (*p* < 0.001) (Table 3).

The highest-scoring items of the FDLQI in the AD group were questions on emotional distress (Q1:1 ± 1), time spent for treatment and house-work (Q7: 1.1 ± 0.9; Q8: 0.9 ± 0.1), and increased household expenditure (Q10: 1.0 ± 0.9).

In total, 186 fully completed DLQI and POEM questionnaires were analyzed in the atopic dermatitis group (24 patients had severe atopic dermatitis, 59 patients had moderate atopic dermatitis, and 103 had mild atopic dermatitis). Our study revealed that the severity of the disease affects patients’ QoL negatively. We found that the mean QoL index for severe atopic dermatitis was 14.25 ± 6.2 (which represents a very large effect); for moderate atopic dermatitis, it was 6.9 ± 4.4 (moderate effect), for mild atopic dermatitis, it was 4.4 ± 4.2 (small effect) (*p* < 0.001), and there were statistically significant differences between groups (*p* < 0.001) (Table 4). Quality of life was more affected for severe AD patients compared with mild and moderate AD patients in almost all aspects of quality of life. Comparing separated questions in mild versus moderate groups, a significant impact was found in the area of symptoms and feelings (Q1; Q2) and personal relationships (Q8).

Analyzing the POEM and FDLQI data (Table 5 FDLQI and POEM), we also found a significant negative effect on families’ QoL, with severe AD having a very strong effect (16.33 ± 7.63, *p* < 0.001), and with moderate and mild AD having a moderate effect (7.64 ± 5.35 and 5.04 ± 5.85) on QoL. When comparing the results between the individual questions and severity groups, we found that all the data were statistically significant, except for those for Q4 (other people’s reaction to skin disease), Q5 (effect on social life), Q6 (effect on recreation/leisure activities), and Q9 (effect on work/studies) in the mild versus moderate AD groups.

## 4. Discussion

Based on a systematic search regarding studies published from 2003 to 2023, this is the first report evaluating QoL in a fairly large sample of Lithuanian pediatric atopic dermatitis patients using the DLQI and FDLQI questionnaires. 

The burden of AD on patients’ QoL has been previously described in studies outside of Lithuania (4.7–10). For summarized results, look at Table 6. The mean DLQI score of our respondents was 6.3 ± 5.6, which reflects a moderate effect of AD on patients’ QoL. This was consistent with the study by Kim et al. [14], which had a CDLQI score of 6.6. Our results were slightly lower than the mean scores of 7.2 reported by Holm et al. [15], 8.7 reported by Ezzedine et al. [16], 7.2 reported by Cheng et al. [17], and 7.6 reported by Sanches-Perez et al. [18], all of which also fall within the moderate effect range. A study conducted by Coutaneau et al. [19] revealed that patients with AD experience poor health-related QoL with a mean DLQI score of 10.2, but in our opinion, this was influenced by a wider spectrum of population age. This study included children and young adult patients with a mean age of population 11.9 ± 13.1 years. 

The Marciniak et al. [20] study evaluated the correlation between both mothers’ and fathers’ QoL, assessed using the FDLQI, and children’s QoL, assessed with the IDLQI. We chose to compare the data from this study with ours because in our study, 95% (288) of those who completed the questionnaires were mothers. In the current study, the mean FDLQI for mothers was 17.1 ± 5.3 points, which indicates a very large effect on families’ QoL. Other studies conducted by other authors also confirm a very large effect on families QoL with the FDLQI index between 11.8 ± 5.8 and 16.45 ± 6.6 [21,22,23,24]. The differences in results could have been influenced by the age of the study populations. The studies that found higher FDLQIs looked at populations of children with an average age of up to 5 years, whereas the study by Cheng et al., whose subjects were of in similar age range to that of ours, estimated a moderate effect on the families’ QoL (mean FDLQI 10 ± 7.8) [17]. However, the mean SCORAD index was lower in a recent study (26.6 ± 19.8) compared with that in other studies. We could not find any studies that compared FDLQI with the severity of illness in accordance with the POEM index. The difference between the SCORAD and POEM scales is that the POEM is a subjective self-assessment tool for determining the severity of disease. We obtained small differences in the analysis of different aspects of the FDLQI between the SCORAD measure and POEM, and it would be useful to extend future studies with this perspective.

**Table 6:** Comparison of QoL studies on patients with atopic dermatitis.

Study	Number of Participants with AD	Age of Patients with AD	Severity Scale or AD, Mean Score ± SD	QoL Scale: Mean Score ± SD	FDLQI Mean Score ± SD
Holm et al., 2016 [9]	*n* = 296	Mean: 26.3 years	SCORAD (children), 29.4 ± 14.0	CDLQI: 7.2 ± 4.6 CDLG/DLQI: 9.7 ± 5.9	-
Kim et al., 2012 [14]	*n* = 415	Mean: 14.5 ± 10.8 years	SCORAD, 15.8 ± 8.4	IDLQI (*n* = 71): 7.7 ± 5.5 CDLQI (*n* = 197): 6.6 ± 6.3 DLQI (*n* = 147): 10.7 ± 7.9	-
Ezzedine et al., 2020 [16]	*n* = 400	12–18 years	POEM (12–14 y.) Mild: 5.6 ± 5.6 Moderate: 12.7 ± 7.0 Severe: 15.9 ± 4.1 POEM (15–17 y.) Mild: 6.7 ± 6.8 Moderate: 12.4 ± 7 Severe: 18.2 ± 6.8	CDLQI: 8.7 ± 7.1 (12–14 y.) DLQI: 12.8 ± 11.1 (15–17 y.)	-
Cheng et al., 2019 [17]	*n* = 323	2–17 years, *n* = 151 ≥18 years, *n* = 172	SCORAD, 26.58 ± 19.77	CDLQI: 7.2 ± 6.8	9.97 ± 7.99
Sanchez-Perez J., Dauden-Tello E., 2012 [18]	*n* = 151	Mean age: 9.4 ± 4.5 years	IGA, Mild: 3.8 ± 3.0 Moderate: 8.8 ± 4.7 Severe: 14.5 ± 8.2	CDLQI: 7.6 ± 5.7	-
Coutaneau et al., 2014 [19]	*n* = 3914	Mean age: 11.9 ± 13.1 years	SCORAD (*n* = 3589), 42.2 ± 17.3	-	-
Marciniak et al., 2017 [20]	*n* = 50	Mean age: 10.2 ± 6.5 months	-	IDLQI 14.1 ± 4.6 (6–26) 14.0	Mothers: 17.1 ± 5.3 Fathers: 14.7 ± 5.8
Kose et al., 2022 [24]	*n* = 122	Mean age: 5.4 ± 2.3 years	SCORAD, 57.6 ± 11	Mean CDLQI 7.3 ± 6.8	14.8 ± 4.7
Cheng et al., 2019 [17]	*n* = 155	Mean age: 5.4 ± 2.3 years	SCORAD, 26.6 ± 19.8	-	9.97 ± 7.99
Pustisek et al., 2016 [23]	*n* = 171	3 months-17 years	SCORAD, 38.7 ± 16.4	-	13.6 ± 6.0
Chernyshov et al., 2014 [22]	*n* = 30	Mean age: 23.7 ± 24.6 months	SCORAD, 40.6 ± 11.3	-	11.8 ± 5.8
Kobusiewicz et al., 2022 [21]	*n* = 88	Mean age: 60.2 ± 56.6 months	SCORAD, 46.6 ± 15.2	-	16.5 ± 6.6

Abbreviations: AD, atopic dermatitis; SD, standard deviation; SCORAD, Scoring for Atopic Dermatitis; POEM, Patient-Oriented Eczema Measure; IDQoL, Infants’ Dermatitis Quality of Life index (IDQoL); CDLQI, Children’s Dermatology Life Quality Index; DLQI, Dermatology Life Quality Index; FDLQI, Family Dermatology Life Quality Index.

Differences in QoL scores between studies reflect both a variation in disease severity and the subjective nature of QoL impairment, which is undoubtedly influenced by many factors including family and peer relationships, gender, social class, ethnicity, education, and previous life experience. There are differences in the population data of the conducted studies, which may have implications for the data analysis, but overall, the data confirm that AD has a negative impact on patients’ and families’ QoL, that this issue needs to be integrated into the treatment plan, and that additional psychological support needs to be offered to patients and their family members. Alternatively, such support may include training programs, cognitive therapy, and others. 

Our study has some limitations. First, although it was conducted on a fairly large sample of atopic dermatitis patients, it lacked equal distribution around the country. Also, our subjects cannot be considered representative of all atopic dermatitis patients, as they were people attending dermatological services. Larger studies on population-based samples possibly involving primary care physicians and combining a cumulative life course impairment study with DLQI assessment are needed. Precision medicine and a knowledge of the factors affecting inflammatory skin disorders (atopic dermatitis; psoriasis), such as internal and external exosomes, can help patients in all aspects of their lives.

## 5. Conclusions

Our study observed a moderate effect of AD on the dermatology-related QoL of patients and of their families. The association between the severity of AD and the effect on patients’ and families’ quality of life was established. Quality of life assessments should be included in the factors that determine treatment choices in many countries’ treatment guides.

## Figures and Tables

**Table 1:** Description of the study populations.

Participants’ Characteristics	Child AD Group	Child Control Group	Parents/Caregivers
Sex, *n* (%)			
Male	125 (51%)	28 (51%)	15 (5%)
Female	122 (49%)	27 (49%)	288 (95%)
Age (years)	6.84 ± 4.43	10.5 ± 3.1	37.26 ± 6.49
Place of residence			
City	N/A	N/A	225 (74%)
Countryside	N/A	N/A	37 (12%)
Suburb	N/A	N/A	33 (11%)
N/A	N/A	N/A	3 (1%)
Marital status			
Married	N/A	N/A	257 (85%)
Cohabiting partner	N/A	N/A	28 (9%)
Divorced	N/A	N/A	11 (4%)
Single	N/A	N/A	4 (1%)
N/A	N/A	N/A	3 (1%)
Family history of atopy	169 (69%) *	24 (47.1%) *	N/A
Food allergy	99 (43%) **	1 (2%) **	N/A
Allergic asthma	28 (12%)	3 (6%)	N/A
Allergic rhinoconjunctivitis	67 (29%) ***	7 (13%) ***	N/A
All three atopic diseases	13 (5%)	-	N/A
First AD symptoms			
<3 months	91 (37%)	N/A	N/A
4–5 months	48 (20%)	N/A	N/A
6–12 months	50 (20%)	N/A	N/A
1–3 years	32 (13%)	N/A	N/A
3–5 years	9 (4%)	N/A	N/A
>5 years	8 (3%)	N/A	N/A

Abbreviations: AD, atopic dermatitis; N/A, not applicable. Note: the numbers marked with * have a *p*-value < 0.048; the numbers marked with ** have a *p*-value < 0.001; the numbers marked with *** have a *p*-value < 0.022.

**Table 2:** QoLI in atopic dermatitis and healthy control patients.

Variable	Mean ± SD AD (*n* = 247)	Mean ± SD Controls (*n* = 50)	*p*-Value
QoLI	6.3 ± 5.6	0.5 ± 1.1	<0.001
QoLI.Q1	1.4 ± 0.9	0.2 ± 0.4	<0.001
QoLI.Q2	1 ± 0.9	0.1 ± 0.4	<0.001
QoLI.Q3	0.6 ± 0.8	0 ± 0.1	<0.001
QoLI.Q4	0.6 ± 0.8	0 ± 0.1	<0.001
QoLI.Q5	0.4 ± 0.8	0 ± 0	<0.001
QoLI.Q6	0.4 ± 0.8	0 ± 0	<0.001
QoLI.Q7	0.4 ± 0.7	0 ± 0.1	<0.001
QoLI.Q8	0.3 ± 0.6	0 ± 0	<0.001
QoLI.Q9	0.6 ± 0.9	0.1 ± 0.3	<0.001
QoLI.Q10	0.6 ± 0.8	0.1 ± 0.2	<0.001

Abbreviations: AD, atopic dermatitis, SD, standard deviation; QoLI, quality of life index, Q-question. Notes: QoLI Q1: how itchy, sore, painful or stinging has your skin been? Q2: how embarrassed or self conscious have you been because of your skin? Q3: how much has your skin interfered with you going shopping or looking after your home? Q4: how much has your skin influenced the clothes you wear? Q5: how much has your skin affected any social or leisure activities? Q6: how much has your skin made it difficult for you to do any sport? Q7: has your skin prevented you from working or studying? Q8: how much has your skin created problems with your partner or any of your close friends or relatives? Q9: how much has your skin caused any sexual difficulties? Q10: how much of a problem has the treatment for your skin been, for example by making your home messy, or by taking up time.

**Table 3:** FDLQI in atopic dermatitis and healthy control patients.

Variable	Mean ± SD AD (*n* = 247)	Mean ± SD Controls (*n* = 50)	*p*-Value
FDQLI	7.05 ± 6.92	2.1 ± 5.9	<0.001
FDLQI_Q1	1 ± 1	0.2 ± 0.6	<0.001
FDLQI_Q2	0.7 ± 0.9	0.2 ± 0.6	<0.001
FDLQI_Q3	0.5 ± 0.8	0.2 ± 0.6	0.004
FDLQI_Q4	0.5 ± 0.8	0.1 ±0.6	<0.001
FDLQI_Q5	0.4 ± 0.8	0.1 ± 0.6	0.014
FDLQI_Q6	0.5 ± 0.9	0.2 ± 0.6	<0.001
FDLQI_Q7	1.1 ± 0.9	0.4 ± 0.7	<0.001
FDLQI_Q8	0.9 ± 0.1	0.2 ± 0.6	<0.001
FDLQI_Q9	0.4 ± 0.8	0.1 ± 0.6	<0.001
FDLQI_Q10	1.0 ± 0.9	0.4 ± 0.7	<0.001

Abbreviations: AD, atopic dermatitis; SD, standard deviation; FDLQI, Family Dermatology Life Quality Index, Q-question. Notes: FDLQI questions: Q1: how much emotional distress have you experienced due to your relative/partner’s skin disease (e.g., worry, depression, embarrassment, frustration?). Q2: has your relative/partner’s skin disease affected your physical well-being (e.g., tiredness, exhaustion, contribution to poor health, sleep/rest disturbance?). Q3: how much has your relative/partner’s skin disease affected your personal relationships with him/her or with other people? Q4: how much have you been having problems with other peoples’ reactions due to your relative/partner’s skin disease (e.g., bullying, staring, need to explain to others about his/her skin problem? Q5: how much has your relative/partner’s skin disease affected your social life (e.g., going out, visiting or inviting people, attending social gatherings?) Q6: how much has your relative/partner’s skin disease affected your recreation/leisure activities (e.g., holidays, personal hobbies, gym, sports, swimming, watching TV? Q7: how much time have you spent on looking after your relative/partner (e.g., putting on creams, giving medicines, or looking after their skin? Q8: how much extra housework have you had to do because of your relative/partner’s skin disease (e.g., cleaning, vacuuming, washing, cooking? Q9: how much has your relative/partner’s skin disease affected your job/study (e.g., need to take time off, not able to work, decrease in the number of hours worked, having problems with people at work? Q10: how much has your relative/partner’s skin disease increased your routine household expenditure (e.g., travel costs, buying special products, creams, cosmetics?

**Table 4:** Comparison of QoL index between severity groups, scored POEM.

QoL Index (IDLQI/CDLQI/DLQI)	POEM Group	Mean ± SD	*p*-Value	*p*-Value: Mild vs. Severe	*p*-Value: Mild vs. Moderate	*p*-Value: Moderate vs. Severe
QoL index	Severe	14.3 ± 6.2	<0.001	<0.001	<0.001	<0.001
	Moderate	6.9 ± 4.4	<0.001	<0.001	<0.001	<0.001
	Mild	4.4 ± 4.2	<0.001	<0.001	<0.001	<0.001
QoL.Q1	Severe	2.5 ± 0.6	<0.001	<0.001	<0.001	<0.001
QoL.Q1	Moderate	1.8 ± 0.7	<0.001	<0.001	<0.001	<0.001
QoL.Q1	Mild	0.9 ± 0.8	<0.001	<0.001	<0.001	<0.001
QoL.Q2	Severe	2.2 ± 0.9	<0.001	<0.001	0.001	<0.001
QoL.Q2	Moderate	1.2 ± 0.9	<0.001	<0.001	0.001	<0.001
QoL.Q2	Mild	0.7 ± 0.7	<0.001	<0.001	0.001	<0.001
QoL.Q3	Severe	1.3 ± 0.9	<0.001	<0.001	0.198	0.001
QoL.Q3	Moderate	0.6 ± 0.8	<0.001	<0.001	0.198	0.001
QoL.Q3	Mild	0.5 ± 0.6	<0.001	<0.001	0.198	0.001
QoL.Q4	Severe	1.2 ± 1.1	0.003	0.014	0.067	0.001
QoL.Q4	Moderate	0.4 ± 0.8	0.003	0.014	0.067	0.001
QoL.Q4	Mild	0.6 ± 0.7	0.003	0.014	0.067	0.001
QoL.Q5	Severe	1.0 ± 1.1	<0.001	<0.001	0.456	<0.001
QoL.Q5	Moderate	0.4 ± 0.8	<0.001	<0.001	0.456	<0.001
QoL.Q5	Mild	0.3 ± 0.6	<0.001	<0.001	0.456	<0.001
QoL.Q6	Severe	1.1 ± 1.1	<0.001	<0.001	0.078	0.001
QoL.Q6	Moderate	0.4 ± 0.8	<0.001	<0.001	0.078	0.001
QoL.Q6	Mild	0.2 ± 0.5	<0.001	<0.001	0.078	0.001
QoL.Q7	Severe	0.9 ± 0.8	<0.001	<0.001	0.279	0.003
QoL.Q7	Moderate	0.4 ± 0.6	<0.001	<0.001	0.279	0.003
QoL.Q7	Mild	0.3 ± 0.6	<0.001	<0.001	0.279	0.003
QoL.Q8	Severe	0.6 ± 0.7	0.012	0.003	0.200	0.065
QoL.Q8	Moderate	0.3 ± 0.5	0.012	0.003	0.200	0.065
QoL.Q8	Mild	0.3 ± 0.6	0.012	0.003	0.200	0.065
QoL.Q9	Severe	1.7 ± 1	<0.001	<0.001	<0.001	<0.001
QoL.Q9	Moderate	0.8 ± 0.9	<0.001	<0.001	<0.001	<0.001
QoL.Q9	Mild	0.4 ± 0.6	<0.001	<0.001	<0.001	<0.001
QoL.Q10	Severe	1.6 ± 0.9	<0.001	<0.001	0.043	<0.001
QoL.Q10	Moderate	0.6 ± 0.9	<0.001	<0.001	0.043	<0.001
QoL.Q10	Mild	0.4 ± 0.6	<0.001	<0.001	0.043	<0.001

Abbreviations: AD, atopic dermatitis; CDLQI, Children Dermatology Life Quality Index; DLQI, Dermatology Life Quality Index; IDLQI, Infants’ Dermatology Life Quality Index; POEM, Patient-Oriented Eczema Measure; SD, standard deviation; Q, question. Notes: QoLI.Q1: how itchy, sore, painful or stinging has your skin been? Q2: how embarrassed or self conscious have you been because of your skin? Q3: how much has your skin interfered with you going shopping or looking after your home? Q4: how much has your skin influenced the clothes you wear? Q5: how much has your skin affected any social or leisure activities? Q6: how much has your skin made it difficult for you to do any sport? Q7: has your skin prevented you from working or studying? Q8: how much has your skin created problems with your partner or any of your close friends or relatives? Q9: how much has your skin caused any sexual difficulties? Q10: how much of a problem has the treatment for your skin been, for example by making your home messy, or by taking up time.

**Table 5:** Comparison of FDLQI between severity groups, scored using POEM.

QoL Index	POEM Group	Mean ± SD	*p*-Value	*p*-Value: Mild vs. Severe	*p*-Value: Mild vs. Moderate	*p*-Value: Moderate vs. Severe
FDLQI	Severe	16.3 ± 7.6	<0.001	<0.001	0.001	<0.001
FDLQI	Moderate	7.6 ± 5.4	<0.001	<0.001	0.001	<0.001
FDLQI	Mild	5 ± 5.9	<0.001	<0.001	0.001	<0.001
FDLQI_Q1	Severe	2.2 ± 0.8	<0.001	<0.001	<0.001	0.001
FDLQI_Q1	Moderate	1.4 ± 0.9	<0.001	<0.001	<0.001	0.001
FDLQI_Q1	Mild	0.6 ± 0.8	<0.001	<0.001	<0.001	0.001
FDLQI_Q2	Severe	1.9 ± 1	<0.001	<0.001	0.001	<0.001
FDLQI_Q2	Moderate	0.9 ± 0.9	<0.001	<0.001	0.001	<0.001
FDLQI_Q2	Mild	0.5 ± 0.7	<0.001	<0.001	0.001	<0.001
FDLQI_Q3	Severe	1.4 ± 1.1	<0.001	<0.001	0.021	<0.001
FDLQI_Q3	Moderate	0.5 ± 0.8	<0.001	<0.001	0.021	<0.001
FDLQI_Q3	Mild	0.3 ± 0.7	<0.001	<0.001	0.021	<0.001
FDLQI_Q4	Severe	1.3 ± 1.1	<0.001	<0.001	0.296	0.001
FDLQI_Q4	Moderate	0.6 ± 0.7	<0.001	<0.001	0.296	0.001
FDLQI_Q4	Mild	0.4 ± 0.6	<0.001	<0.001	0.296	0.001
FDLQI_Q5	Severe	1.2 ± 1.3	<0.001	<0.001	0.516	<0.001
FDLQI_Q5	Moderate	0.2 ± 0.6	<0.001	<0.001	0.516	<0.001
FDLQI_Q5	Mild	0.3 ± 0.7	<0.001	<0.001	0.516	<0.001
FDLQI_Q6	Severe	1.7 ± 1.1	<0.001	<0.001	0.610	<0.001
FDLQI_Q6	Moderate	0.4 ± 0.7	<0.001	<0.001	0.610	<0.001
FDLQI_Q6	Mild	0.4 ± 0.7	<0.001	<0.001	0.610	<0.001
FDLQI_Q7	Severe	2.1 ± 0.8	<0.001	<0.001	0.000	<0.001
FDLQI_Q7	Moderate	1.3 ± 0.8	<0.001	<0.001	0.000	<0.001
FDLQI_Q7	Mild	0.8 ± 0.7	<0.001	<0.001	0.000	<0.001
FDLQI_Q8	Severe	1.5 ± 1.1	0.001	<0.001	0.033	0.032
FDLQI_Q8	Moderate	1 ± 1	0.001	<0.001	0.033	0.032
FDLQI_Q8	Mild	0.7 ± 1	0.001	<0.001	0.033	0.032
FDLQI_Q9	Severe	1.3 ± 1.2	<0.001	<0.001	0.616	0.001
FDLQI_Q9	Moderate	0.3 ± 0.6	<0.001	<0.001	0.616	0.001
FDLQI_Q9	Mild	0.3 ± 0.7	<0.001	<0.001	0.616	0.001
FDLQI_Q10	Severe	1.8 ± 1	<0.001	<0.001	0.003	0.004
FDLQI_Q10	Moderate	1.1 ± 0.8	<0.001	<0.001	0.003	0.004
FDLQI_Q10	Mild	0.8 ± 0.9	<0.001	<0.001	0.003	0.004

Abbreviations: AD, atopic dermatitis; FDLQI, Family Dermatology Life Quality Index; POEM, Patient-Oriented Eczema Measure; standard deviation; Q, question. Notes: FDLQI questions: Q1: how much emotional distress have you experienced due to your relative/partner’s skin disease (e.g., worry, depression, embarrassment, frustration?). Q2: has your relative/partner’s skin disease affected your physical well-being (e.g., tiredness, exhaustion, contribution to poor health, sleep/rest disturbance?). Q3: how much has your relative/partner’s skin disease affected your personal relationships with him/her or with other people? Q4: how much have you been having problems with other peoples’ reactions due to your relative/partner’s skin disease (e.g., bullying, staring, need to explain to others about his/her skin problem?). Q5: how much has your relative/partner’s skin disease affected your social life (e.g., going out, visiting or inviting people, attending social gatherings?); Q6: how much has your relative/partner’s skin disease affected your recreation/leisure activities (e.g., holidays, personal hobbies, gym, sports, swimming, watching TV?). Q7: how much time have you spent on looking after your relative/partner (e.g., putting on creams, giving medicines, or looking after their skin?). Q8: how much extra housework have you had to do because of your relative/partner’s skin disease (e.g., cleaning, vacuuming, washing, cooking?). Q9: how much has your relative/partner’s skin disease affected your job/study (e.g., need to take time off, not able to work, decrease in the number of hours worked, having problems with people at work?). Q10: how much has your relative/partner’s skin disease increased your routine household expenditure (e.g., travel costs, buying special products, creams, cosmetics?).

## Data Availability

The raw data supporting the conclusions of this article will be made available by the authors on request.
